# Alternative Perspectives on Impact: The Potential of ALMs and Altmetrics to Inform Funders about Research Impact

**DOI:** 10.1371/journal.pbio.1002003

**Published:** 2014-11-25

**Authors:** Adam Dinsmore, Liz Allen, Kevin Dolby

**Affiliations:** Wellcome Trust, London, United Kingdom

## Abstract

More evidence of the meaning and validity of ALMs and altmetrics, coupled with greater consistency and transparency in their presentation, would enable research funders to explore their potential value and identify appropriate use cases.

Medical research charities fund more than a third of all publicly funded medical research in the United Kingdom. In the financial year of 2012–2013, the UK's charitable spending on medical research totalled £1.3 billion [1]. The Wellcome Trust is the second-highest spending charitable foundation in the world [2] and contributed almost half of this amount by awarding £538 million in support of biomedical research, development of medical technologies, and the medical humanities [3].

With such large investments in play, funders are naturally keen to understand and learn from the impact of the work they support. At the Wellcome Trust, the sort of impact we might expect can vary widely depending on the funding programme, from the discovery of a novel biomarker for childhood pneumonia [4], to the generation of a map of H7N9 infection [5] to assist epidemic preparedness in China, to heightened public engagement with science through the development of a video game based on the principles of Mendelian genetics [6].

Attempts to capture such a wide range of research impacts require a toolbox of methods and approaches to track the reach, use, and reuse of research outputs such as journal articles, datasets, and software. Expert peer review has long been and will continue to be an important component of judgements of the quality of research. Metrics, when used properly, can both inform and complement that process [7]; bibliometric analysis of research publications and their citation impact has been used for many years to provide a proxy measure of the impact of research within the scholarly literature [8]. Though we know that the impacts of research extend far beyond the academic literature [9]—to clinicians, policy makers, educators and the general public—accessible means of gauging this impact have not been so readily available.

## “Alternative” Impact and the New Metrics

However, times are changing. The migration of the academic literature from paper journals to online platforms has brought about the emergence of a new class of alternative metrics, usually referred to as article-level metrics (ALMs) or altmetrics. Shema et al. [10] define these new metrics as “web-based metrics for the impact of scholarly material, with an emphasis on social media outlets as sources of data”. In addition to academic citations, these metrics aggregate views, downloads, discussions, and recommendations of research outputs across the scholarly web [11], as well as citations in nonacademic communications such as policy documents [12], patent applications, and clinical guidelines. Though typically termed article-level metrics, ALMs and altmetrics are often equally applicable to other research outputs, including datasets, code, and software [13].

ALMs and altmetrics offer research funders greater intelligence regarding the use and reuse of research, both among traditional academic audiences and stakeholders outside of academia. The new metrics can provide evidence of the reach, uptake, and diffusion of research, which is valuable to funders looking to explore alternative routes to impact. While conventional citation data will continue to play a major role in research evaluation, the new metrics have the potential to provide a valuable complement to the insights revealed by traditional bibliometric indicators.

## How Are Funders Using ALMs/Altmetrics Currently?

The Wellcome Trust—among other research funders—is exploring the potential value of ALMs/altmetrics to support organisational learning and funding strategy. For example, the Trust is investigating how alternative metrics might be used to detect early engagement with research in the policy sphere; Box 1 details the case of Jim McCambridge's [14] review of industry submissions to a government consultation on alcohol policy, which generated considerable social media attention among policy makers following its publication in *PLOS Medicine* in 2013. This attention was visible long before academic citations to the paper had begun to appear and came largely from stakeholders outside of academia whose engagement would usually remain invisible to conventional bibliometrics.

**Box 1. Behind the Metrics: Social Media Activity as a Proxy of Engagement in the Policy Sphere**

Early detection of engagement with research by policy makers can allow the Wellcome Trust to explore how we can best reach nonacademic audiences and better understand routes from research to integration in policy and practise. Illuminating these processes may provide vital means of bringing greater efficiency to the research pipeline.

Most ALM providers allow users to investigate the meaning behind the numbers, at least on an ad hoc basis. For example, clicking the Twitter logo on the metrics tab of this article will produce a list of tweets that link back to it (presuming anyone has judged it interesting enough to tweet by the time you read this). This can allow for the discovery of possible evidence of impact that might otherwise remain invisible.

In 2013, a study led by Jim McCambridge [14], a Wellcome Trust Research Career Development Fellow, examined industry submissions to a 2008 Scottish government consultation on alcohol policy. The resultant article was published in *PLOS Medicine* and alleged that many submissions misrepresented research findings so as to support policies favoured by the alcohol industry.

**Figure 1:**
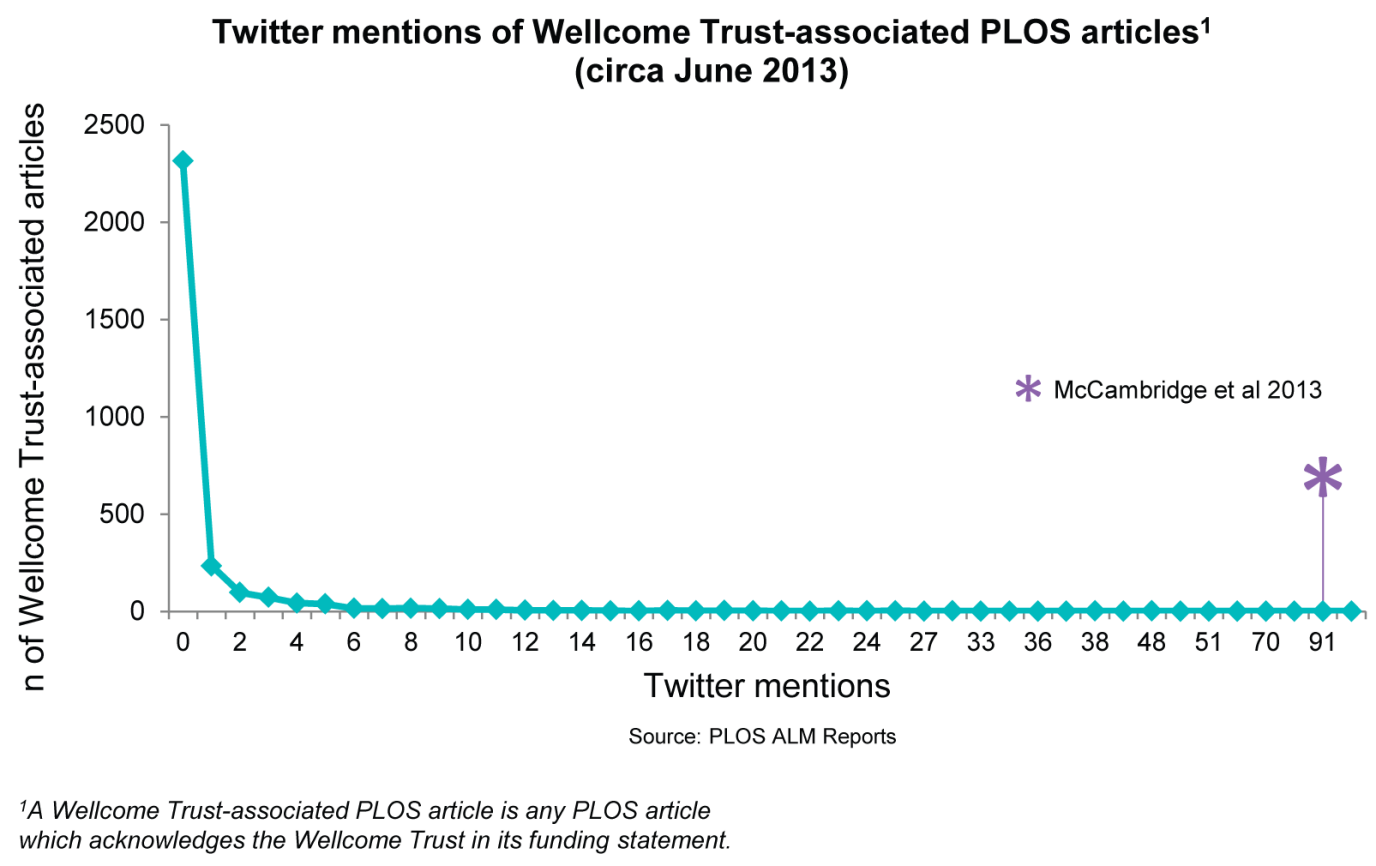
Tweets discussing Wellcome Trust-associated PLOS articles c. June 2013.

Though the paper remained uncited for three months following its publication, it was tweeted at four times the average rate for *PLOS Medicine* articles published in 2013 and 70 times the average rate of all Wellcome Trust–associated articles published across the family of PLOS journals (see Figure 1). Many of the accounts tweeting about the paper belonged to key influencers, including members of the European Parliament, international nongovernmental organisations (NGOs), and a sector manager for Health, Nutrition, and Population at the World Bank, suggesting that a study with an apparently parochial focus had nonetheless had a rapid influence all over the world.

Such information could be valuable to researchers and funders looking for effective routes to policy makers. Of course, significant attention in the social media space does not mean that the research is necessarily high quality or will lead to major impact, but greater intelligence on the diffusion and reach of work that is associated with subsequent policy impact would help research funders think about their strategies for uptake and engagement.

ALMs and altmetrics may also be useful to the research funding process. Most research funders award funding based on the quality of an idea and the track record of an applicant, of which publication history is typically an important part. ALMs may help provide context for peer reviewers on the use, reach, and influence of scholarly work both inside and out of the academic sphere, perhaps reducing any reliance on assumptions that research published in more “prestigious” journals is necessarily of higher impact. Alternative metrics of impact may be particularly beneficial to junior researchers, especially those who may not have had the opportunity to accrue a sufficient body of work to register competitive scores on traditional indicators, or those researchers whose particular specialisms seldom result in key author publications.

## Meaning, Validity, and the Science of Science

ALMs/altmetrics present an opportunity for more debate about our definition and understanding of impact, but this needs to be evidence based. Much of the existing literature examines correlations between altmetrics and academic citations, often with significant results. For example, Thelwall et al. [15] found statistically significant associations between metric scores and citations for all metrics for which there was sufficient evidence, suggesting that altmetrics may have some potential as a means of predicting academic impact soon after publication.

More research is required to explore the value and validity of the new metrics as part of the toolkit available to those who monitor research impact. There are a whole host of interesting questions concerning ALMs/altmetrics, the answers to which would help funders think about the impact of the research they have funded and how to do science more effectively. For example, can academics use social media to speed up the uptake of research into policy and practice? Do undergraduate students typically read the articles they bookmark in Mendeley? How might we normalise ALM/altmetric scores such that valid comparisons can be drawn between research outputs published at different times and in different scholarly disciplines?

## Consistency in the ALM/Altmetric Ecosystem

One of the challenges faced by the ALM/altmetric community is consistency of view. ALM/altmetric scores can either be presented as raw counts of (for example) page views, downloads, and social media mentions or by combining several indicators into a single composite score. Differences in presentation notwithstanding, there is currently little consistency between the scores provided by the various vendors of ALMs/altmetrics, and it can therefore be difficult for users to decide which numbers are most trustworthy and appropriate to their particular use case. This is not peculiar to the new metrics—counts of academic citations made available by Scopus, Web of Science, and Google Scholar also tend to differ [16]—but greater uniformity is nonetheless required if the new metrics are to be seen as reliable indicators of impact.

These apparent discrepancies may result from differences in technical approach between vendors, each of whom must decide precisely how to define units such as page views, downloads, and social media mentions. For example, is it more appropriate to count the times an article is viewed or the number of unique readers? Does a tweet to a blog post that reviews an article among several others count as much as a tweet to the paper itself? Increased transparency in regards to these definitions would allow users to analyse and interpret scores with greater confidence. That said, effective impact tracking is only possible when research outputs can be clearly linked to the researchers who produce them and the funding that supports them. The aims of funders and vendors alike would also be served by greater integration of persistent identifiers in the metadata that accompany research outputs across the scholarly web, which would allow outputs to be clearly linked to the researchers who produce them and the funders that support them [17].

## Bringing Greater Efficiency to the Scientific Enterprise

There is currently an appetite for greater insight into the value of new metrics concerning scholarly work, as shown by the recent call for evidence relating to the use of metrics in research assessment and management by the Higher Education Funding Council for England (HEFCE). The call is part of a wider review of the use of metrics in research assessment, with a particular focus on how and where metrics might play a role in future iterations of the UK's Research Excellence Framework (REF) [18]. If the review finds that research metrics have developed sufficiently in the six years since the previous review [19], ALMs/altmetrics may provide a useful suite of complementary measures for the next REF, likely to take place in 2020, thereby reducing the considerable administrative burden currently placed on researchers and institutions.

Given that national governments are now taking an interest in alternative views of impact, there is a great opportunity for all stakeholders in the research process to support efforts to better understand what these metrics tell us and how they might be used to the benefit of science. This includes greater engagement between funders and ALM platform developers to ensure that funders' requirements to track and assess research are accommodated whenever possible. If we work together, the resultant platforms and metrics should increase our ability to understand the development and impact of research and help bring efficiencies to the scientific enterprise.
